# Differential expression of the miR-17-92 cluster and miR-17 family in breast cancer according to tumor type; results from the Norwegian Women and Cancer (NOWAC) study

**DOI:** 10.1186/s12967-019-2086-x

**Published:** 2019-10-03

**Authors:** Line Moi, Tonje Braaten, Khalid Al-Shibli, Eiliv Lund, Lill-Tove Rasmussen Busund

**Affiliations:** 1 Institute of Medical Biology, UiT The Arctic University of Norway, Tromsø, Norway; 2 Department of Clinical Pathology, University Hospital of North Norway, Tromsø, Norway; 3 Institute of Community Medicine, UiT The Arctic University of Norway, Tromsø, Norway; 4 Department of Pathology, Nordland Hospital, Bodø, Norway; 5 Cancer Registry of Norway, Oslo, Norway

**Keywords:** Breast cancer, MicroRNA, miR-17-92-cluster, miR-106b-25 cluster, miR-17-family, NOWAC

## Abstract

**Background:**

MicroRNAs (miRNAs) are promising biomarkers due to their structural stability and distinct expression profile in various cancers. We wanted to explore the miRNA expression in benign breast tissue and breast cancer subgroups in the Norwegian Women and Cancer study.

**Methods:**

Specimens and histopathological data from study participants in Northern Norway diagnosed with breast cancer, and benign tissue from breast reduction surgery were collected. Main molecular subtypes were based on surrogate markers; luminal A (ER+ and/or PR+, HER2− and Ki67 ≤ 30%), luminal B (ER+ and/or PR+, HER2− and Ki67 > 30% or ER+ and/or PR+ and HER2+), HER2 positive (ER− and PR− and HER2+) and triple-negative (ER−, PR− and HER2−). RNA was extracted from formalin-fixed paraffin-embedded (FFPE) tissue, and miRNAs were successfully analyzed in 102 cancers and 36 benign controls using the 7th generation miRCURY LNA microarray containing probes targeting all human miRNAs as annotated in miRBASE version 19.0. Validation with RT-qPCR was performed.

**Results:**

On average, 450 miRNAs were detected in each sample, and 304 miRNAs were significantly different between malignant and benign tissue. Subgroup analyses of cancer cases revealed 23 miRNAs significantly different between ER+ and ER− tumors, and 47 miRNAs different between tumors stratified according to grade. Significantly higher levels were found in high grade tumors for miR-17-5p (p = 0.006), miR-20a-5p (p = 0.007), miR-106b-5p (p = 0.007), miR-93-5p (p = 0.007) and miR-25-3p (p = 0.015) from the paralogous clusters miR-17-92 and miR-106b-25. Expression of miR-17-5p (p = 0.0029), miR-20a-5p (p = 0.0021), miR-92a-3p (p = 0.011) and miR-106b-5p (p = 0.021) was significantly higher in triple-negative tumors compared to the rest, and miR-17-5p and miR-20a-5p were significantly lower in luminal A tumors.

**Conclusions:**

miRNA expression profiles were significantly different between malignant and benign tissue and between cancer subgroups according to ER− status, grade and molecular subtype. miRNAs in the miR-17-92 cluster and miR-17 family were overexpressed in high grade and triple-negative tumors associated with aggressive behavior. The expression and functional role of these miRNAs should be further studied in breast cancer to explore their potential as biomarkers in diagnostic pathology and clinical oncology.

## Background

Breast cancer is a leading cause of cancer-related deaths. Annually, approximately 2.09 million women worldwide are diagnosed with breast cancer whereas an estimated 627.000 women die of the disease [1]. Breast cancer incidence has been increasing over the last decades in the Western world, also in Norway [2]. Mortality rates are falling, leaving an increasing number of women alive with a history of the disease, but also exposed to risk of complications and side-effects due to treatment and with a life-long risk of relapse. There is a need for simple, safe and informative diagnostic tools to better identify the breast cancer tumors with the most aggressive behavior and to diagnose and treat the disease before distant metastases have been established and the disease is beyond curability.

MicroRNAs (miRNAs) are short single-stranded RNAs built up of 18-22 nucleotides after processing of the pri-miRNA by the nuclear RNase III protein Drosha and sequentially cleavage of the hairpin-shaped precursor-miRNA by the RNase III Dicer in the cells’ cytoplasm [3]. MiRNAs are important regulators of gene expression at the post-transcriptional level, usually by either inhibiting translation or inducing mRNA degradation through incomplete or complete binding to a complementary sequence in the 3′ untranslated region (UTR) of their target mRNAs. It is well established that miRNAs are involved in carcinogenesis, invasion and metastasis [4, 5] and display distinct profiles in cancer [6]. Further, miRNAs have properties that make them promising as biomarkers. They can be detected in blood, partly in extracellular vesicles known as exosomes, and in other body fluids such as urine and saliva [7, 8]. MiRNAs are very stable structures and can tolerate freezing and thawing [9]. Further, if miRNAs in blood could give information on the phenotype or aggressiveness of a given tumor, there is a possibility that easily obtained samples could give information on malignant disease, both at the time of primary diagnosis and in the metastatic setting [10].

The Norwegian Women and Cancer study (NOWAC) is a prospective study which started in 1991 and includes 172,000 Norwegian women aged 30–70 years randomly sampled from the Norwegian Central Person Registry. The study is based on questionnaires with information on variables of importance to breast cancer risk such as lifestyle, use of oral contraceptives, hormone replacement therapy, reproductive history and family history of breast cancer. From 2003 the study was expanded to include blood samples for whole-genome expression profiling (the NOWAC postgenome cohort) [11]. 49,633 samples of peripheral blood have been collected. Through linkage to the Cancer Registry of Norway, women in the NOWAC postgenome cohort with a diagnosis of breast cancer have been identified.

The aim of this pilot study was to explore the miRNA expression profile in breast cancer tumors from the NOWAC postgenome cohort and to search for miRNAs that are significantly different in tumor tissue compared to benign breast tissue and could be detected in formalin-fixed paraffin-embedded (FFPE) tissue, collected as part of routine diagnostics. Further, we wanted to identify miRNAs that are differently expressed in tumors with different aggressiveness and prognosis with special focus on high grad tumors and the triple-negative breast cancers.

## Methods

### Patient material and tumor classification

The 108 patients included in this pilot study were all participants in the NOWAC postgenome cohort living in Northern Norway and diagnosed with breast cancer in the years 2004–2010. Diagnostic biopsies and breast cancer surgery were performed at the University Hospital of North Norway or the Nordland Central Hospital. As benign controls, FFPE tissues from 44 women undergoing breast reduction surgery were included in the study. The controls were born in the same time period as the NOWAC participants (1943–1957) and underwent surgery in the same time period. Archived FFPE tissue blocks were retrieved from the pathology labs at the University Hospital of North Norway and the Nordland Central Hospital together with the corresponding hematoxylin and eosin slides.

Histopathological data such as histological type, grade, size and lymph node status were collected from the original pathology reports, but reevaluated and completed according to updated criteria by the breast pathologist in charge. Tumor grade was assessed based on gland formation, nuclear pleomorphism and mitotic count, based on the criteria modified by Elston and Ellis [12]. Immunohistochemical (IHC) analyses of estrogen receptor (ER), progesterone receptor (PR) and HER2 were performed on the needle biopsies from tumor taken at the time of diagnosis, as part of routine diagnostics. Cut-off value for ER positivity was ≥ 1% and for PR ≥ 10%. A HER2 IHC score of 3+ was considered positive, a score of 0–1+ negative whereas a score of 2+ would lead to further assessment of HER2 status by silver in situ hybridization (SISH). HER2 gene amplification was considered present if HER2/chromosome 17-ratio was > 2.2. IHC staining for the proliferation marker Ki67 was done on histological slides of tumor tissue from the primary surgery to differentiate between luminal A and luminal B tumors. Ki67 expression was evaluated in at least 500 tumor cells in the most proliferative active parts of tumor and reported as the percentage of positive tumor cell nuclei.

The main molecular breast cancer subtypes were defined based on clinical grouping and immunohistochemical staining of ER, PR, HER2 and Ki67 as recommended by the St Gallen International Expert Consensus and previous publications [13, 14] as follows: luminal A (ER+ and/or PR+, HER2− and Ki67 ≤ 30%), luminal B (ER+ and/or PR+, HER2− and Ki67 > 30% or ER+ and/or PR+ and HER2+), HER2 positive (ER− and PR− and HER2+) and triple-negative (ER−, PR− and HER2−). The term triple-negative cancer was preferred over basal-like breast cancer since subgrouping was done as part of clinical, treatment-oriented classification and was based on receptor status alone. Most basal-like cancers are triple-negative, but triple-negative breast cancers are found to be genetically heterogeneous and are generally not considered synonymous with basal-like tumors [15], although considerable overlap has been demonstrated [16].

### RNA extraction and microarray procedures

A trained pathologist selected the most representative tumor areas on histological slides and tissue cores from the corresponding areas of the FFPE blocks were collected for extraction of total RNA using the RecoverAll Total Nucleic Acid Isolation kit (Life Technologies, Grand Island, NY, USA). Exiqon (Vedbaek, Denmark) performed the microarray hybridization and analyses as a bought service. In short, RNA quality and quantity was assessed using the NanoDrop 1000 spectrophotometer (Thermo Fisher Scientific, Wilmington, DE), and 250 ng total RNA from samples and reference was labeled with Hy3™ and Hy5™ fluorescent label (Exiqon, Vedbaek, Denmark), respectively, using the miRCURY LNA™ microRNA Hi-Power Labeling Kit (Exiqon, Vedbaek, Denmark). The Hy5™-labeled reference RNA contained an equal aliquot of all RNA species included in the study. The Hy3™-labeled samples and Hy5™-labeled reference RNA were mixed and hybridized to the 7th generation miRCURY LNA miRNA array (Exiqon) which contains capture probes targeting identified miRNAs in human, mouse, rat and their related viral sequences as annotated in miRBASE. According to Exiqon, the array contained 3100 capture probes covering 94% of the human miRNAs in miRBASE version 19.0. The hybridization was done using a Tecan HS4800 hybridization station (Tecan, Austria) and the microarray slides were stored in an ozone free environment after hybridization. The slides were scanned using the Agilent G2565BA Microarray Scanner System (Agilent technologies Inc., USA) and the image analysis was carried out using the ImaGene 9.0 software (BioDiscovery Inc., USA). The quantified signals were background corrected and normalized using quantile normalization method. The detection threshold was calculated as 1.2 times the 25th percentile of the overall signal intensity of the individual slides. MiRNAs with intensities above threshold in < 20% of the samples were removed from the final dataset used for the expression analyses to ensure that the expression analyses were done on miRNAs which were expressed in enough samples to be of biological relevance [17]. However, all miRNAs, independently of the percentage of samples with detectable expression, were included in a screening analysis to explore if any one miRNA was uniquely expressed in any of the tumor subgroups.

### RT-qPCR validation

**Fig. 1:**
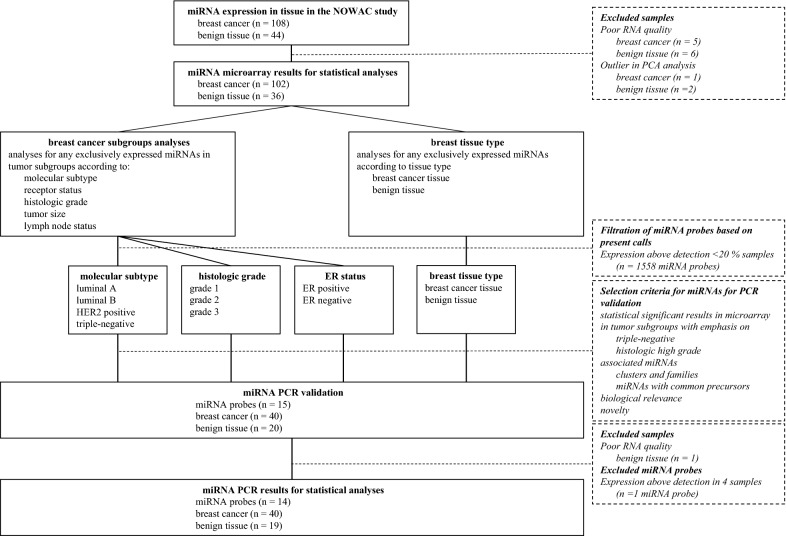
Study schema for the analyses of miRNA expression in tissues. The schema illustrates the work flow, the statistical analyses and the subgroup analyses performed on data from miRNA microarray and PCR in the study

To validate the microarray miRNA analyses, RT-qPCR was performed by Exiqon on RNA purified from 40 of the tumor samples and 20 of the benign breast tissue controls included in the study. Due to the clinical relevance of the molecular subtypes and the scientific focus on the triple-negative breast cancers, the tumor samples were randomly selected within each molecular subtype so that there would be about ten cases in each subgroup. 15 of the miRNAs from the microarray analysis were selected for validation. PCR-validation was done after the initial statistical analyses on the miRNA expression levels from the microarray had been performed. MiRNAs with highly significant differences between tissue types and/or tumors subgroups according to tumor grade and receptor status with special focus on the triple-negative breast cancers, were selected for validation. Associated miRNAs, such as miRNA clusters, -family or -strands from a common precursor with a coordinated regulation of expression were given priority over individual miRNAs. However, four single miRNAs of potential interest for future studies were also included, based on known biological functions or potential for novel findings, based on the microarray. The following miRNAs were analyzed using PCR: the let-7 family members let-7b-5p and let-7c, the two miR-126-strands miR-126-5p and miR-126-3p, the miR-143-145 cluster members miR-143-3p and miR-145-5p, and the miR-17-92 cluster/miR-17-family members miR-17-5p, miR-20a-5p, miR-92a-3p, miR-106b-5p and miR-93-5p. In addition, the well described miR-155-5p and miR-146a-5p were included in the PCR-analyses. Finally, the less known miRNAs miR-3182 and miR-3164 were included in the PCR-analyses for exploratory purposes. The design of the study and a schematic overview of the analyses done is presented in Fig. 1.

In short, the Qiagen miRNeasy FFPE kit was used to extract RNA from FFPE tissue cores according to the manufacturer’s instructions (Qiagen, Hilden, Germany). 10 ng RNA was reverse transcriped using the miRCURY LNA Universal RT microRNA PCR, Polyadenylation and cDNA synthesis kit (Exiqon) before PCR-reactions were performed on 100× diluted cDNA using ExiLENT SYBR Green master mix. The amplification was done in 384 well plates in a Light Cycler 480 Real-Time PCR System (Roche, Basel, Switzerland) where all reverse transcription reactions were done in duplicates. The Normfinder software was used by Exiqon to find the most suitable reference miRNAs based on stable expression across the data set. Of the suitable reference miRNAs, miR-664a-3p was detected in all samples in concentrations comparable to the target miRNAs and could be used for normalization. Normalized expression values for each miRNA were calculated based on the PCR quantification cycle (Cq) and the average of the normalizer detected in all samples using the formula: normalized Cq = average Cq (all samples) − assay Cq (sample).

### Statistics

The miRNA microarray and PCR expression data were analysed using the Linear Models for Microarray and RNA-Seq Data (Limma) package in R. Moderated F-statistics were applied. *p*-values were corrected for multiple testing by controlling the false discovery rate using the method of Benjamini & Hochberg. Descriptive statistics, non-parametric tests and correlation analysis, using Pearson correlation, were performed using Stata, version 14 (StataCorp LLC, Texas, USA).

## Results

### Patient and tumor characteristics

The women included in the NOWAC study are all born in the period 1943–1957. The mean age at breast cancer diagnosis in our study was 57.6 years (range 47–65 years) whereas the benign breast tissue controls were collected from women with a mean age at surgery of 57.4 years (range 47–66 years). Of the 108 breast cancer tumors, 81 tumors (75%) were hormone receptor positive, 20 tumors (18.5%) were HER2 positive of which 11 tumors (10.2%) were triple-positive (ER+, PR+, HER2+), and 16 cases (14.8%) had triple-negative tumors. 28 of the tumors (25.9%) had histologic characteristics of aggressive breast cancer with low differentiation, cellular atypia and high mitotic activity, corresponding to histologic grade 3. Molecular subclassification of tumors, based on surrogate markers, demonstrated 80 tumors (74.1%) to be of the luminal type, whereas 9 (8.3%) and 16 tumors (14.8%) ended up as HER2 positive and triple-negative, respectively. The patient material and tumor characteristics are presented in Table 1.

**Table 1:** Patient characteristics and clinicopathological variables for the study population and samples included in PCR validation

Characteristic	NOWAC pilot study			
	Study population		PCR analyses	
	Cases N (%)	Controls N (%)	Cases N (%)	Controls N (%)
Study subjects	108 (100)	44 (100)	40 (100)	20 (100)
Age (years)				
≤ 50	7 (6.5)	1 (2.3)	1 (2.5)	1 (5.0)
> 50	101 (93.5)	43 (97.7)	39 (97.5)	19 (95.0)
Tumor grade				
1	34 (31.5)	–	7 (17.5)	–
2	42 (38.9)	–	17 (42.5)	–
3	28 (25.9)	–	16 (40.0)	–
Unknown	4 (3.7)		0 (0)	
Tumor size (mm)				
0–10	23 (21.3)	–	2 (5.0)	–
10–20	50 (46.3)	–	24 (60.0)	–
> 20	34 (31.5)	–	14 (35.0)	–
Unknown	1 (0.9)		0 (0)	
Lymph node status				
N0	73 (67.6)	–	30 (75.0)	–
N+	34 (31.5)	–	10 (25.0)	–
Unknown	1 (0.9)	–	0 (0)	–
Hormone receptor status				
ER/PR positive	81 (75.0)	–	23 (57.5)	–
ER/PR negative	27 (25.0)	–	17 (42.5)	–
Unknown	0 (0)	–	0 (0)	–
HER2 receptor status				
HER2 positive	20 (18.5)	–	15 (37.5)	–
HER2 negative	86 (79.6)	–	25 (62.5)	–
Unknown	2 (1.9)	–	0 (0)	–
Molecular subtype				
Luminal A	58 (53.7)	–	12 (30.0)	–
Luminal B	22 (20.4)	–	11 (27.5)	–
HER2 positive	9 (8.3)	–	7 (17.5)	–
Triple-negative	16 (14.8)	–	10 (25.0)	–
Unknown	3 (2.8)		0 (0)	
Histological type				
Infiltrating carcinoma NST	92 (85.2)	–	35 (87.5)	–
Lobular carcinoma	10 (9.3)	–	3 (7.5)	–
Tubular	3 (2.8)	–	0 (0)	–
Other	3 (2.8)	–	2 (5.0)	–

### MiRNA expression levels in malignant versus normal breast tissue

Of a total of 108 cases of invasive breast cancer cases in this pilot study, five specimens did not have satisfactory RNA quality and were not included in the microarray, and one specimen was identified as an extreme outlier in the unsupervised analyses of the miRNA results using principal component analysis (PCA), leaving 102 breast cancer specimens for final analysis. Six of the 44 benign breast surgery specimens had RNA quality of unsatisfactory quality and two benign tissue controls were identified as outliers after unsupervised microarray analysis, resulting in 36 benign tissue controls in the final statistical analyses.

**Fig. 2:**
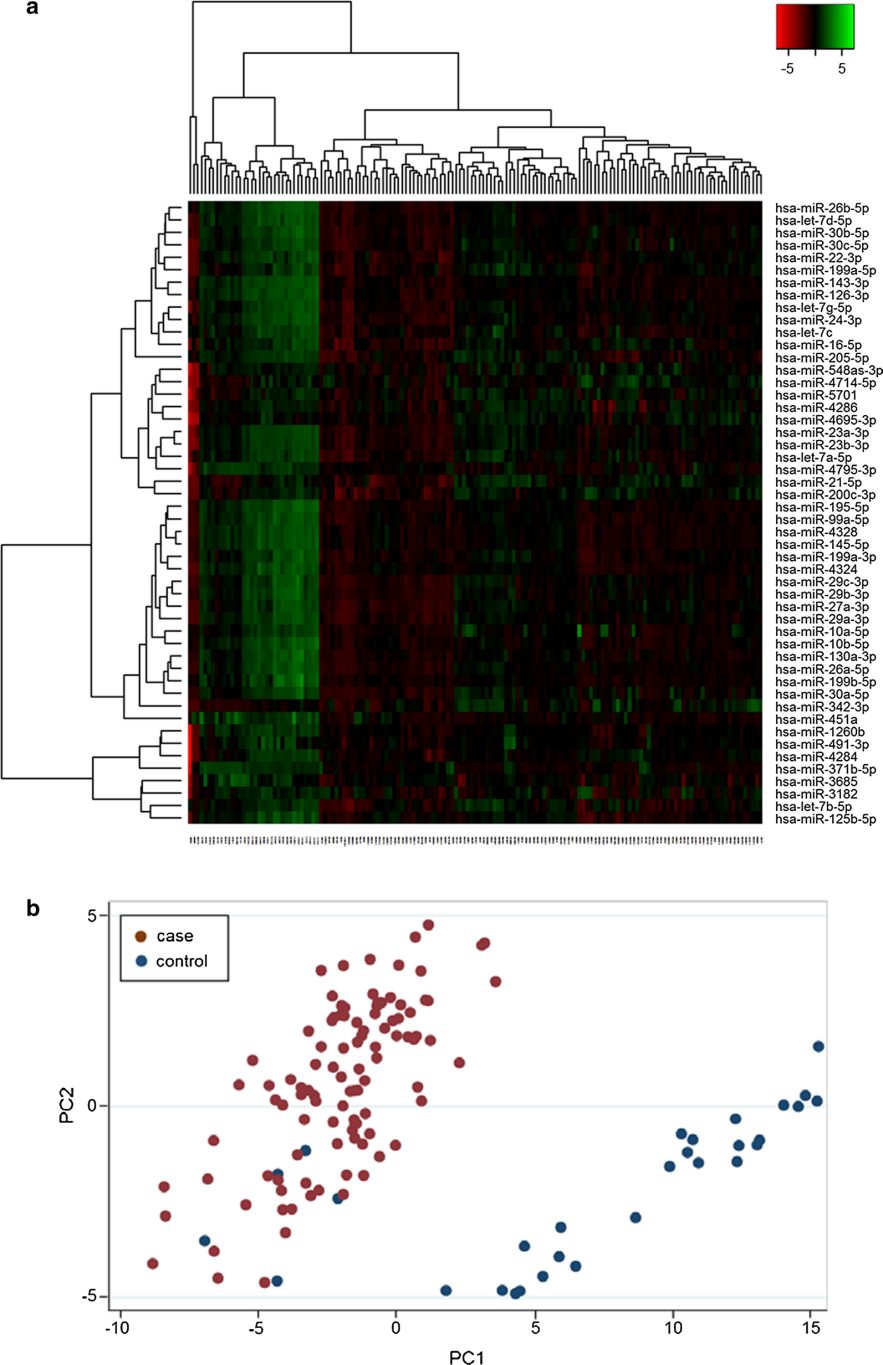
Heat map and principal component analysis of miRNAs with greatest variability between cases and controls. **a** Heat map diagram with unsupervised hierarchical clustering is presented for all samples and the top 50 miRNAs with the largest variation across all samples. Each row represents a miRNA and each column represents a sample. The color scale illustrates the relative expression level of a miRNA across all samples: red color represents an expression level below the mean, green color expression above the mean. **b** Plot of the principal component analysis performed on all samples and on the 50 miRNAs with the largest variation across all samples. The normalized log-transformed Hy3 values have been used for the analysis

Screening analyses of all detected probes did not demonstrate any miRNAs that were uniquely expressed in any of the tissue types or breast cancer subgroups. For further expression analyses, 1558 probes were discarded due to intensities above threshold in less than 20% of the samples. Noteworthy, the samples contained similar levels of detectable miRNAs indicating comparable sample quality. On average, 450 different miRNAs were detected above threshold in each sample. 304 miRNAs demonstrated significantly different expression levels in tumor tissue compared to normal tissue. Heat map and PCA demonstrated that the miRNAs in normal tissue and tumor samples clustered according to their biological group (Fig. 2). This indicates that differences between the groups are the largest contributors to variation in miRNA expression and underlines that the miRNA profile in malignant and benign breast tissue is significantly different.

**Fig. 3:**
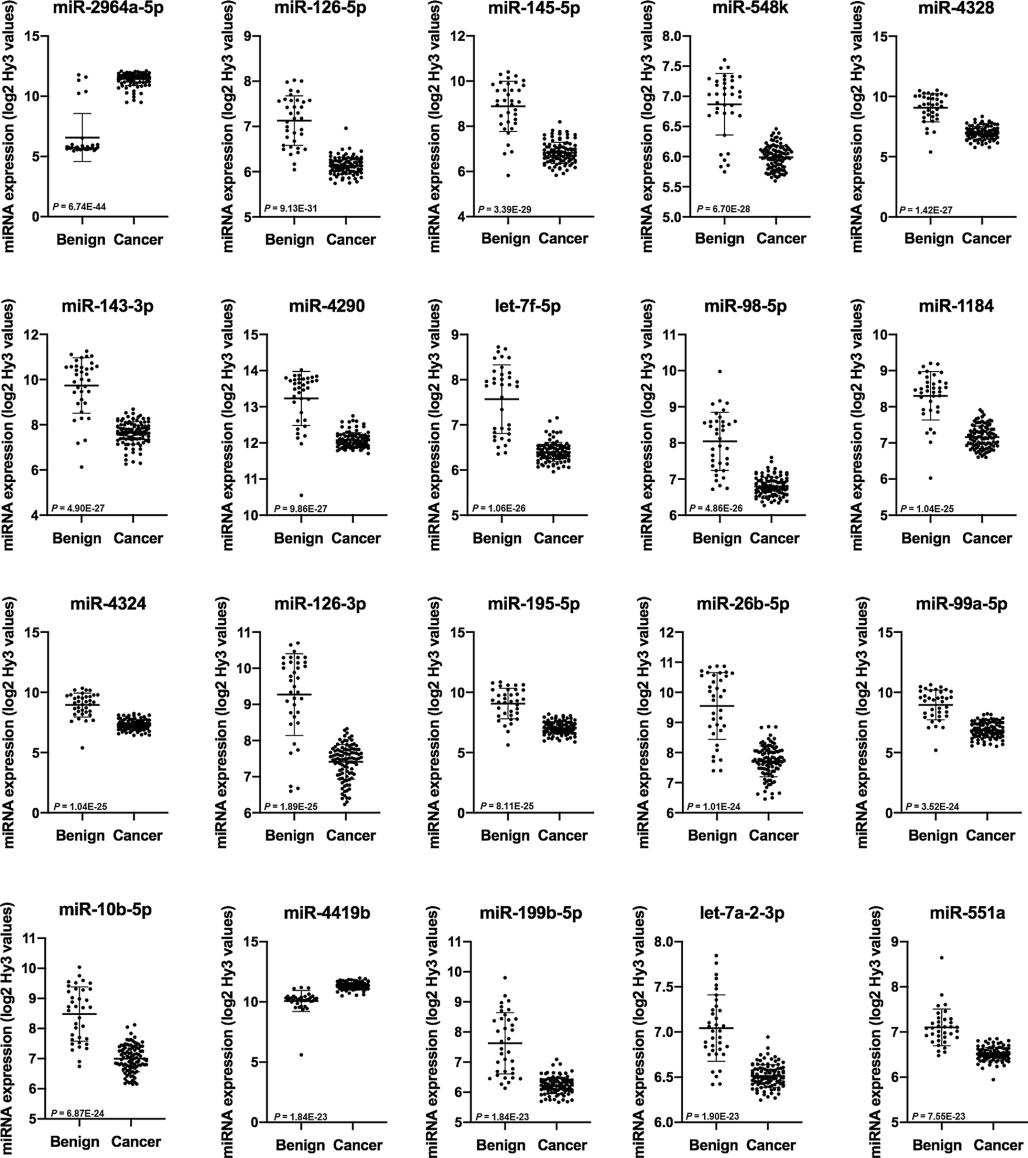
The 20 most differently expressed miRNAs in breast cancer and benign tissue, based on p-values. The scatterplots show the expression of the miRNAs in 36 benign tissue controls and 102 breast cancer tumors analyzed by microarray. Mean, standard deviation and false discovery rate (FDR) adjusted *p*-values are presented. E indicates exponential number

Scatterplots of the 20 most significantly differently expressed miRNAs in malignant breast tumors compared to benign breast tissue, based on p-values, are shown in Fig. 3. Noteworthy, of these 20 miRNAs, 18 miRNAs were downregulated in cancer compared to benign breast tissue.

Further, the calculated log fold change (logFC) between malignant and benign tissue add**s** information on the probable biological relevance of the observed difference in miRNA expression. The 20 most upregulated and the 20 most downregulated miRNAs in cancer, based on the logFC-values, are listed in Table 2.

**Table 2:** The most up- and downregulated miRNAs in cancer compared to benign tissue, sorted by logFC

MiRNA	LogFC cancer versus benign	p*
miR-2964a-5p	4.859	6.74E−44
miR-4419b	1.249	1.84E−23
miR-3164	1.146	2.22E−12
miR-K12-5-5p	1.143	2.90E−12
miR-221-5p	0.924	1.51E−17
miR-585	0.920	1.80E−11
miR-342-3p	0.865	1.07E−07
miR-766-5p	0.846	1.77E−10
miR-4478	0.828	1.03E−21
miR-338-5p	0.815	2.64E−12
miR-21-5p	0.703	8.74E−04
miR-342-5p	0.667	3.27E−13
miR-149-3p	0.660	1.46E−08
miR-5196-3p	0.650	6.37E−20
miR-182-5p	0.644	9.59E−08
miR-4644	0.592	8.30E−08
miR-224-3p	0.588	2.81E−11
miR-4768-5p	0.586	2.13E−12
miR-4778-3p	0.565	5.08E−17
miR-4417	0.559	5.72E−05

miR-451a	− 2.647	1.89E−18
miR-143-3p	− 2.096	4.90E−27
miR-99a-5p	− 2.093	3.52E−24
miR-4328	− 2.090	1.42E−27
miR-145-5p	− 2.061	3.39E−29
miR-205-5p	− 2.027	1.45E−12
miR-195-5p	− 2.023	8.11E−25
miR-125b-5p	− 1.970	8.70E−14
miR-126-3p	− 1.862	1.89E−25
miR-26b-5p	− 1.837	1.01E−24
miR-4324	− 1.651	1.04E−25
miR-29a-3p	− 1.592	3.44E−12
miR-10b-5p	− 1.483	6.87E−24
miR-30b-5p	− 1.452	1.03E−15
let-7d-5p	− 1.450	6.25E−21
miR-130a-3p	− 1.406	4.80E−19
miR-199b-5p	− 1.400	1.84E−23
miR-29b-3p	− 1.339	1.56E−14
miR-199a-3p	− 1.316	2.13E−13
miR-24-3p	− 1.287	7.37E−18

Log fold change (logFC) values are calculated, based on the log2 transformed intensity values (Hy3) by microarray analyses, comparing breast cancer to benign tissue controls. The 20 most upregulated and the 20 most downregulated miRNAs, sorted by highest and lowest logFC, respectively, are presented

* False discovery rate (FDR) adjusted *p*-value. E indicates exponential number

### MiRNA expression levels according to breast cancer subtypes

**Fig. 4:**
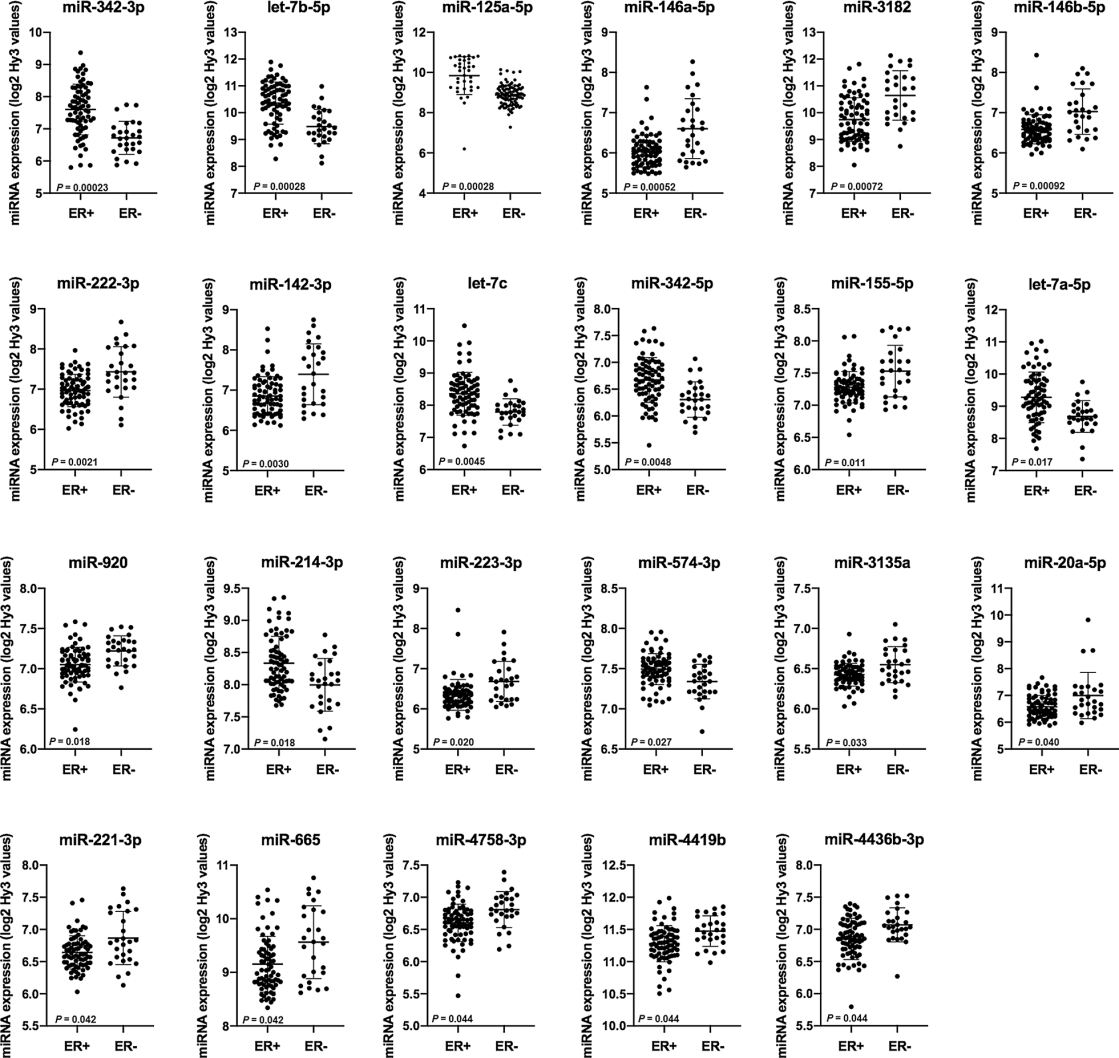
MiRNAs demonstrating significantly different expression according to estrogen receptor status. The scatterplots show the expression of miRNAs in estrogen receptor positive and estrogen receptor negative breast cancers. Mean, standard deviation and false discovery rate (FDR) adjusted *p*-values are presented

The expression of miRNAs in breast cancer stratified according to receptor expression, histologic grade, molecular subtype, tumor size and lymph node status was explored, using moderated F-statistics and the Benjamini & Hochberg correction for multiple comparisons on microarray miRNA expression data. The analysis revealed that 23 of the detected miRNAs were significantly different between ER+ and ER− tumors (Table 3 and Fig. 4).

**Table 3:** MiRNAs demonstrating significantly different expression according to estrogen receptor status

miRNA	ER positive breast cancer		ER negative breast cancer		logFC	p*
	Mean	SD	Mean	SD		
miR-342-3p	7.60	0.79	6.72	0.51	0.881	2.29E−4
let-7b-5p	10.36	0.79	9.48	0.64	0.878	2.76E−4
miR-125a-3p	9.01	0.46	8.50	0.42	0.514	2.76E−4
miR-146a-5p	6.04	0.40	6.60	0.74	− 0.561	5.16E−4
miR-3182	9.74	0.82	10.64	0.92	− 0.900	7.20E−4
miR-146b-5p	6.59	0.36	7.03	0.56	− 0.442	9.16E−4
miR-222-3p	6.97	0.40	7.43	0.63	− 0.464	0.0021
miR-142-3p	6.85	0.48	7.39	0.76	− 0.540	0.0030
let-7c	8.36	0.67	7.79	0.41	0.564	0.0045
miR-342-5p	6.67	0.42	6.30	0.33	0.365	0.0048
miR-155-5p	7.28	0.24	7.53	0.40	− 0.251	0.011
miR-let-7a-5p	9.28	0.79	8.68	0.49	0.595	0.017
miR-920	7.05	0.22	7.22	0.19	− 0.171	0.018
miR-214-3p	8.33	0.42	8.00	0.41	0.337	0.018
miR-223-5p	6.35	0.38	6.68	0.50	− 0.332	0.020
miR-574-3p	7.50	0.19	7.34	0.21	0.154	0.027
miR-3135a	6.42	0.14	6.54	0.22	− 0.126	0.033
miR-20a-5p	6.58	0.42	7.00	0.86	− 0.420	0.040
miR-221-3p	6.64	0.26	6.87	0.41	− 0.223	0.042
miR-665	9.15	0.52	9.56	0.68	− 0.410	0.042
miR-4758-3p	6.60	0.29	6.81	0.28	− 0.205	0.044
miR-4419b	11.28	0.28	11.47	0.24	− 0.195	0.044
miR-4436b-3p	6.85	0.32	7.07	0.26	− 0.219	0.044

Expression levels are given as the mean with standard deviation (SD) of log2 transformed intensity values (Hy3) by microarray analyses

* False discovery rate (FDR) adjusted *p*-value. E indicates exponential number

**Fig. 5:**
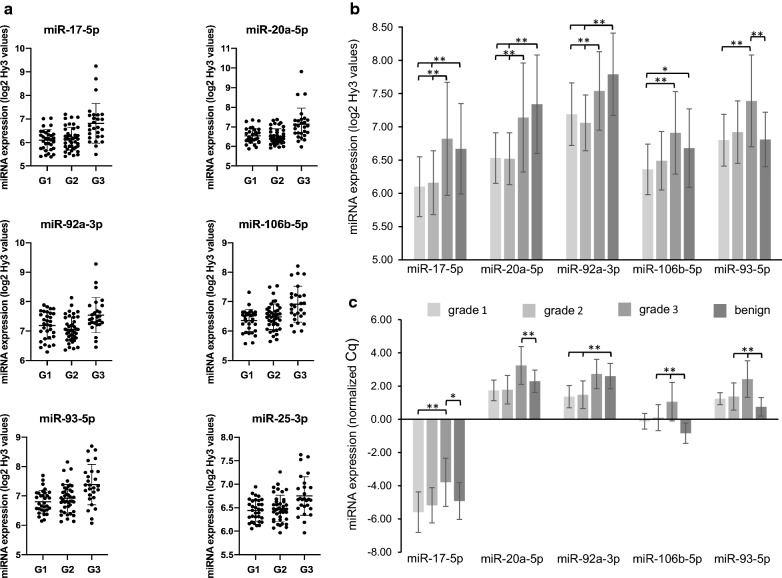
Expression levels of miRNAs in the miR-17-92 and miR-106b-25 clusters according to histologic grade. **a** Scatterplots show the expression of miRNAs in breast cancer cases according to their histologic grade: grade 1 (G1), grade 2 (G2) or grade 3 (G3). Means and standard deviations from microarray analyses are presented. *p*-values for comparisons are presented in **b**. **b** MiRNA expression in breast cancer cases and in benign tissue controls is presented with microarray expression levels given as mean (standard deviation) of log2 transformed Hy3 intensity values. *False discovery rate (FDR) adjusted *p*-value < 0.05, **p-value < 0.01. **c** MiRNA expression in breast cancers and in benign tissue analyzed by qPCR. Expression levels are presented as mean (standard deviation) of normalized PCR quantification cycle (Cq) values based on the average of the normalizer assay detected in all samples and the formula: normalized Cq = average Cq (all samples) − assay Cq (sample). Negative PCR expression values indicate lower expression levels compared to the normalizer. *False discovery rate (FDR) adjusted *p*-value < 0.05, **p-value < 0.01

A total of 47 miRNAs demonstrated significantly different expression between tumors stratified according to histologic grade. Noteworthy, among the six miRNAs with the most significantly different expression according to grade, two miRNAs in the miR-17-92 cluster and two miRNAs in the paralogue miR-106b-25 cluster were represented: miR-17-5p (p = 0.006), miR-20a-5p (p = 0.007), miR-106b-5p (p = 0.007), and miR-93-5p (p = 0.007). Also the cluster members miR-25-3p and miR-92a-3p demonstrated significantly different expression according to grade. Subgroup analyses with contrast tests demonstrated that all of these miRNAs in the miR-17-92 and miR-106b-25 clusters were significantly higher expressed in high grade tumors (Table 4 and Fig. 5a, b).

**Table 4:** Expression of miRNAs in the miR-17-92 and miR-106b-25 clusters according to histologic grade

MiRNA	Tissue	Grade	n	Mean (SD)	Contrasts	p*
miR-17-5p	Benign	–	36	6.67 (0.68)		
	Cancer	All	102	6.32 (0.66)	Cancer vs benign	0.014
	Cancer	1	32	6.10 (0.45)	Grade 1 vs 2 and 3	0.0040
	Cancer	2	41	6.16 (0.48)	Grade 1 and 2 vs 3	2.45E−5
	Cancer	3	27	6.82 (0.85)	Grade 1 vs 3	6.84E−5
miR-20a-5p	Benign	–	36	7.34 (0.74)		
	Cancer	All	102	6.69 (0.60)	Cancer vs benign	1.23E−6
	Cancer	1	32	6.54 (0.38)	Grade 1 vs 2 and 3	0.014
	Cancer	2	41	6.52 (0.39)	Grade 1 and 2 vs 3	2.45E−5
	Cancer	3	27	7.14 (0.82)	Grade 1 vs 3	8.50E−5
miR-92a-3p	Benign	–	36	7.79 (0.62)		
	Cancer	All	102	7.23 (0.52)	Cancer vs benign	1.23E−6
	Cancer	1	32	7.19 (0.47)	Grade 1 vs 2 and 3	0.27
	Cancer	2	41	7.06 (0.42)	Grade 1 and 2 vs 3	0.00037
	Cancer	3	27	7.55 (0.59)	Grade 1 vs 3	0.0068
miR-106b-5p	Benign	–	36	6.68 (0.59)		
	Cancer	All	102	6.56 (0.51)	Cancer vs benign	0.24
	Cancer	1	32	6.36 (0.38)	Grade 1 vs 2 and 3	0.0019
	Cancer	2	41	6.49 (0.44)	Grade 1 and 2 vs 3	6.84E−5
	Cancer	3	27	6.91 (0.61)	Grade 1 vs 3	6.84E−5
miR-93-5p	Benign	–	36	6.81 (0.41)		
	Cancer	All	102	7.00 (0.56)	Cancer vs benign	0.095
	Cancer	1	32	6.80 (0.39)	Grade 1 vs 2 and 3	0.0032
	Cancer	2	41	6.92 (0.47)	Grade 1 and 2 vs 3	6.84E−5
	Cancer	3	27	7.39 (0.69)	Grade 1 vs 3	8.50E−5
miR-25-3p	Benign	–	36	6.63 (0.33)		
	Cancer	All	102	6.54 (0.33)	Cancer vs benign	0.19
	Cancer	1	32	6.44 (0.23)	Grade 1 vs 2 and 3	0.013
	Cancer	2	41	6.48 (0.28)	Grade 1 and 2 vs 3	0.00016
	Cancer	3	27	6.75 (0.41)	Grade 1 vs 3	0.00043

Expression levels are given as the mean (standard deviation) of log2 transformed intensity values (Hy3) by microarray analyses

* False discovery rate (FDR) adjusted *p*-value. E indicates exponential number

The statistical results when analyzing miRNA expression in high grade tumors, indicated that the miR-17-92 and miR-106b-25 clusters were highly interesting. Further, the triple-negative tumors are of special clinical interest due to their aggressive behavior and lack of specific, targeted treatment. When comparing miRNA expression in these tumors compared to the rest, the expression of miR-17-5p (p = 0.0029), miR-20a-5p (p = 0.0021), miR-92a-3p (p = 0.011) and miR-106b-5p (p = 0.021) was significantly higher in triple-negative breast cancers (Table 5).

**Table 5:** Expression of the miR-17-92 cluster and paralogues in triple-negative tumors compared to others, using microarray

MiRNA	Triple-negative tumors		Not triple-negative tumors		logFC	p*
	Mean	SD	Mean	SD		
miR-17-5p	6.80	1.10	6.22	0.50	0.581	0.0029
miR-20a-5p	7.16	1.05	6.60	0.42	0.566	0.0021
miR-92a-3p	7.55	0.75	7.17	0.44	0.384	0.011
miR-106b-5p	6.84	0.72	6.50	0.45	0.341	0.021
miR-93-5p	7.23	0.76	6.96	0.51	0.270	0.094
miR-25-3p	6.66	0.41	6.51	0.31	0.147	0.10

Expression levels are given as the mean (standard deviation) of log2 transformed intensity values (Hy3) by microarray analyses

* False discovery rate (FDR) adjusted *p*-value

**Fig. 6:**
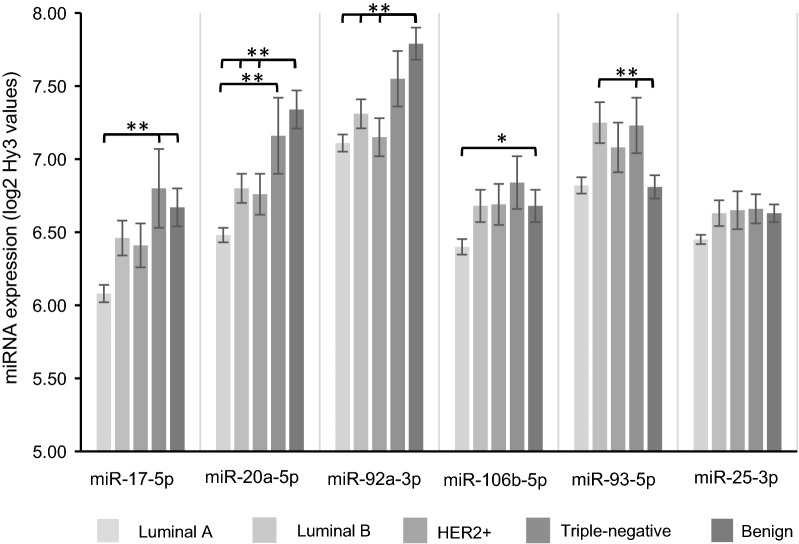
Expression levels of miRNAs in the miR-17-92 and miR-106b-25 clusters according to molecular subtype. MiRNA expression in breast cancer and benign tissue is presented with microarray expression levels given as mean (standard error) of log2 transformed intensity values (Hy3). *False discovery rate (FDR) adjusted *p*-value < 0.05, **p-value < 0.01

Further, overall and contrast tests comparing the miRNA levels in individual molecular subgroups with correction for multiple comparisons were performed. We found that miR-17-5p and miR-20a-5p were significantly different between luminal A and triple-negative tumors (p = 0.001 for both miRNAs), borderline significant between luminal A and luminal B cancers for both miR-17-5p and miR-20a-5p with FDR-adjusted p-values of 0.06 and 0.07, respectively, but not between any of the other molecular subgroups or for any of the other miRNAs in the miR-17-92 and miR-106b-5p clusters (Fig. 6). Further, both miR-17-5p (p = 0.0030) and miR-20a-5p (p = 0.0029) were lower in luminal A tumors compared to the rest.

The expression of 209 miRNAs was significantly different in tumors according to size, using 20 mm in diameter as cutoff (Additional file 1: Table S1). Finally, there were no significant differences in expression of any miRNA in tumors stratified according to HER2 status or lymph node metastasis.

### PCR validation

**Fig. 7:**
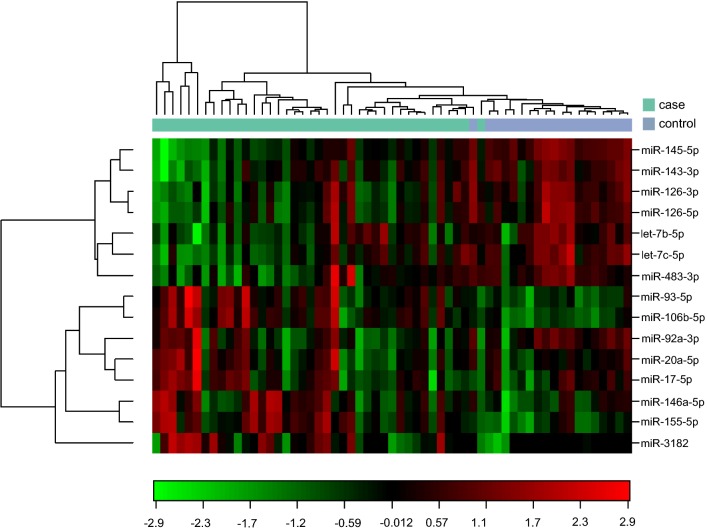
Heat map diagram with two-way hierarchical clustering of miRNAs and samples analyzed by PCR. Each row in the diagram represents one miRNA, and each column represents one sample. The miRNA clustering tree is shown on the left. The color scale illustrates the relative expression level of a miRNA across all samples with red color indicating expression level above the mean, green color expression lower than the mean

14 of the 15 miRNAs selected for PCR validation were successfully quantitated in FFPE tissue, using qPCR (Fig. 7 and Table 6). The previously functionally undescribed miR-3164 was significantly differently expressed in benign versus malignant breast tissue using microarray. However, miR-3164 was only detectable and in very low expression levels in four tumor samples by PCR, which could be due to very low expression levels or suboptimal probes, and was therefore not included in further analyses.

**Table 6:** MiRNA expression in breast cancer and benign breast tissue, measured by PCR

miRNA	Breast cancer	Benign tissue	logFC	p*
	Mean ± SD	Mean ± SD		
let-7b-5p	3.41 ± 1.11	4.28 ± 1.05	− 0.87	0.0094
let-7c	2.54 ± 0.99	3.84 ± 0.90	− 1.30	4.76E−5
miR-126-3p	2.98 ± 1.04	4.24 ± 0.79	− 1.26	5.42E−5
miR-126-5p	− 2.05 ± 1.00	− 0.86 ± 0.80	− 1.18	6.19E−5
miR-143-3p	2.84 ± 1.16	4.18 ± 0.81	− 1.35	6.19E−5
miR-145-5p	3.31 ± 1.53	5.98 ± 0.69	− 2.67	1.79E−8
miR-155-5p	− 0.54 ± 1.37	− 2.23 ± 1.11	1.69	5.04E−5
miR-146a-5p	− 0.48 ± 1.36	− 1.22 ± 1.08	0.74	0.048
miR-3182	− 7.24 ± 1.71	− 9.17 ± 1.43	1.93	0.028
miR-17-5p	− 4.70 ± 1.45	− 4.92 ± 1.11	0.22	0.60
miR-20a-5p	2.36 ± 1.18	2.29 ± 0.67	0.069	0.81
miR-92a-3p	1.96 ± 1.03	2.60 ± 0.76	− 0.65	0.025
miR-106b-5p	0.45 ± 1.03	− 0.84 ± 0.61	1.29	3.81E−5
miR-93-5p	1.76 ± 1.03	0.75 ± 0.56	1.01	0.00031

Expression levels are presented as mean (standard deviation) of normalized PCR quantification cycle (Cq) values based on the average of the normalizer assay detected in all samples and the formula: normalized Cq = average Cq (all samples) − assay Cq (sample). Negative PCR expression values indicate lower expression levels compared to the normalizer

* False discovery rate (FDR) adjusted *p*-value. E indicates exponential number

**Fig. 8:**
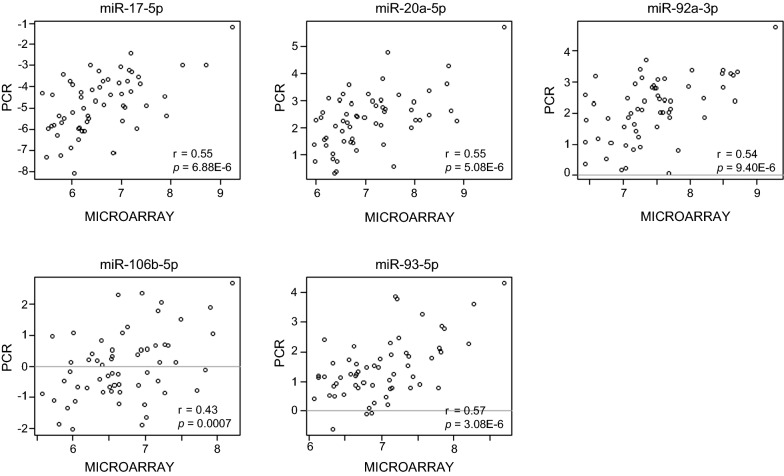
Scatterplots comparing expression levels of individual miRNAs analyzed by microarray and PCR. Expression levels from microarray analyses, given as log2 transformed intensity values (Hy3), are plotted against normalized PCR expression for the different miRNAs. Pearson correlation coefficients (r) and *p*-values are presented in the figure

PCR analyses validated the results from the microarray experiment. Let-7b-5p, miR-146a-5p, miR-3182, let-7c and miR-155-5p were among the miRNAs demonstrating significantly different expression according to ER status in the microarray. Comparison between ER positive and ER negative cancers as measured by PCR, validated the results, demonstrating a logFC of 0.87 for let-7b-5p (p = 0.029), logFC − 1.57 for miR-146a-5p (p = 0.00014), logFC − 0.99 for miR-3182 (p = 0.028), logFC 0.88 for let-7c (p = 0.029) and a logFC of − 1.64 for miR-155-5p (p = 0.00012). Higher levels of the miR-17-92 cluster members were detected in high grade tumors with significant difference between miR-17-5p expression in grade 1 and grade 3 tumors (Fig. 5c). As for microarray, PCR-analyses found significantly higher levels of miR-17-5p (p = 0.0025), miR-20a-5p (p = 0.0053) and miR-92a-3p (p = 0.0073) in triple-negative breast cancers compared to the rest, and the miRNA expression levels tended to be lower in luminal A tumors compared to others, although the adjusted p-values were not significant (Table 7). Importantly, Pearson correlation coefficient demonstrated strong and significant correlations between the microarray and qPCR data for all the 14 detectable miRNAs. Scatterplots comparing microarray and PCR expression levels for the analyzed miR-17-92 and miR-106b-25 cluster members are presented in Fig. 8.

**Table 7:** Expression of miRNAs in the miR-17-92 cluster and paralogues according to molecular subtype, using PCR

MiRNA	Molecular subtype	Mean (SD)	Contrasts	p*
miR-17-5p	Luminal A	− 5.49 (0.73)	Luminal A vs others	0.066
	Luminal B	− 4.93 (1.43)	Luminal B vs others	0.65
	HER2+	− 5.03 (1.70)	HER2+ vs others	0.58
	Triple-negative	− 3.26 (0.95)	Triple-negative vs others	0.0025
miR-20a-5p	Luminal A	1.72 (0.68)	Luminal A vs others	0.066
	Luminal B	2.21 (1.18)	Luminal B vs others	0.67
	HER2+	2.15 (1.19)	HER2+ vs others	0.65
	Triple-negative	3.45 (1.03)	Triple-negative vs others	0.0053
miR-92a-3p	Luminal A	1.44 (0.74)	Luminal A vs others	0.070
	Luminal B	1.75 (1.00)	Luminal B vs others	0.58
	HER2+	1.89 (0.97)	HER2+ vs others	0.85
	Triple-negative	2.87 (0.90)	Triple-negative vs others	0.0073
miR-106b-5p	Luminal A	− 0.014 (0.55)	Luminal A vs others	0.12
	Luminal B	0.30 (1.11)	Luminal B vs others	0.65
	HER2+	0.54 (1.44)	HER2+ vs others	0.91
	Triple-negative	1.10 (0.84)	Triple-negative vs others	0.070
miR-93-5p	Luminal A	1.20 (0.73)	Luminal A vs others	0.070
	Luminal B	1.78 (0.97)	Luminal B vs others	0.93
	HER2+	1.85 (1.21)	HER2+ vs others	0.91
	Triple-negative	2.37 (1.03)	Triple-negative vs others	0.085

Expression levels are presented as mean (standard deviation) of normalized PCR quantification cycle (Cq) values based on the average of the normalizer assay detected in all samples and the formula: normalized Cq = average Cq (all samples) − assay Cq (sample). Negative PCR expression values indicate lower expression levels compared to the normalizer

* False discovery rate (FDR) adjusted *p*-value for contrast tests comparing miRNA expression levels in one molecular subtype of cancers to the other cancers grouped together. E indicates exponential number

## Discussion

This study has explored the miRNA expression profile in breast cancer in the NOWAC study. The distribution of tumor characteristics such as receptor status, histologic grade and molecular subtype is as expected based on findings in larger epidemiological studies and the collected national data in the Norwegian Cancer Registry, again underlining that the NOWAC study population is a representative cohort of the Norwegian female population in this age-group [18].

Noteworthy, we found the miRNA expression profile to be significantly different in benign and malignant breast tissue, as illustrated by the principal component analysis. Of the 20 most differentially expressed miRNAs in microarray, presented in Fig. 3, only two miRNAs demonstrated higher expression levels whereas 18 miRNAs demonstrated lower expression levels in tumor compared to benign breast tissue. Of 14 miRNAs quantitated by PCR, 12 miRNAs were significantly differently expressed in breast cancer and benign tissue, of which seven miRNAs had significantly lower expression in breast cancer tissue. Tumors have often been found to have reduced levels of mature miRNA which could be explained by defects in their biogenesis, for instance through loss of key proteins in their synthesis such as DICER, epigenetic silencing through mechanisms such as promoter hypermethylation and/or genetic loss of miRNA loci [5, 19].

Among the most differentially expressed miRNAs, we found downregulation in tumor tissue of miRNAs such as miR-143-3p and miR-145-5p in the miR-143/145 cluster, miR-10b-5p, miR-99a and let-7a-2-3p which is in line with other studies comparing malignant and benign breast tissue [20–25]. Among the 20 most deregulated miRNAs, we observed significantly higher levels of miR-4419b and miR-2964a-5p. MiR-4419b has been found to be upregulated in small cell tumors of the esophagus with rapid relapse after surgery [26] and miR-2964a in pancreatobiliary adenocarcinoma [27], but they are not functionally described in breast cancer. However, as established by miRNA profiling across cancer types, many of the deregulated miRNAs are common in different malignancies.

Analyses of the breast cancer cases revealed significant differences between cancer subtypes. ER is of special interest in breast cancer as a prognostic and predictive marker, and we found 23 miRNAs to be significantly different according to ER status. Among these, miR-155 has previously been shown to be upregulated in breast cancer compared to normal tissue and upregulated in ER− compared to ER+ tumors [22, 24, 28], as verified in our study. Noteworthy, miR-342-3p, which we found to be most significantly different with higher expression in ER+ compared to ER− tumors, has also been found by others to be strongly associated with ER+ status and to predict ER+ receptor status [29, 30].

Interestingly, when using an agnostic approach and exploring the miRNA expression profile in breast cancer tissue in an epidemiological study using microarray, four of the six miRNAs found to be most significantly different between tumors of different grade, were miR-17-5p, miR-20a-5p, miR-106b-5p and miR-93-5p, belonging to the miR-17-92 cluster and its paralogue miR-106b-25, and all members of the miR-17 family. Further analyses demonstrated that these miRNAs were significantly higher in tumors with high histologic grade and triple-negative status. Other studies have also found higher levels of miRNAs in these clusters in the most aggressive breast cancers using fresh-frozen tumor tissue and a bead-based flow cytometric miRNA expression method, analyzing on a smaller number of miRNAs than in our study [28]. Calvano Filho et al. found higher levels of miRNAs in the miR-17-92 cluster and miR-17 family in selected triple-negative tumors compared to luminal A breast cancers, using RT-PCR [31]. MiR-18a and -18b have been shown to have higher expression levels in ER− compared to ER+ tumors, and to directly target ERα [32].

Of note, when comparing the clustered miRNAs’ expression in breast cancer to benign tissue, we found significantly higher expression of miR-106b-5p and miR-93-5p in breast cancer tissue compared to benign tissue using PCR; by using microarray we found no significant differences. Higher levels of miR-17 family members such as miR-93, miR-25 and miR-106b in cancer have also been demonstrated in other studies using deep sequencing, microarray and/or PCR [33–35]. Several of these studies were smaller than ours and used tissue adjacent to the breast tumor as benign tissue controls. As in our study, using microarray, others have also found members of the miR-17-92 cluster and miR-17 family such as miR-17-5p, miR-20a-5p and miR-92a-3p to be downregulated in solid cancers compared to benign tissue [19, 24]. We could, however, only validate this result for miR-92a-3p using PCR; for miR-17-5p and miR-20a-5p no significant difference was observed. Noteworthy, in the PCR-assays, a relative larger proportion of the cancers was triple-negative compared to the entire study cohort included in the microarray, underlining that differences between breast cancer subgroups could influence comparisons between cancer and benign tissue. Although our study includes more tumor and benign tissue samples compared to many other studies, our study material is still small, and the results must be interpreted with caution. Further, when performing expression analyses on tissue, one must be aware that the tissue cores contain other cellular elements contributing to the RNA pool such as immune cells, endothelial cells and fibroblasts, where malignant tissue would be expected to be more heterogenic compared to benign tissue. Hence, differences in miRNA expression between malignant and benign tissue could also, in part, be attributed to differences in tissue composition where tumor heterogeneity could influence the results [36].

Still, our results from breast cancer subgroup analyses, using both microarray and PCR, point to the miR-17-92 cluster and miR-17-family as overexpressed in aggressive breast tumors. The miR-17-92-cluster is located on chromosome 13 in the locus of the non-protein coding gene *MIR17HG* (miR-17-92 cluster host gene) and was first identified as the gene “chromosome 13 open reading frame 25” (*C13orf25*) found to be amplified in human B-cell lymphoma [37]. The cluster is transcribed as a polycistronic primary transcript that give rise to six mature miRNAs: miR-17, miR-18a, miR-19a, miR-19b, miR-20a, and miR-92a-1 [38, 39]. Transcriptional regulation of *C13orf25* can be part of the molecular basis for the coordinated expression of cluster members as observed in our study. A correlation in expression of the individual miRNAs in the miR-17-92 cluster [29] and also correlation of expression of the miR-106b-25 cluster members and their host gene *MCM7* on chromosome 7 has been shown [28]. The miR-17-92 cluster has been shown to be regulated by the transcription factor and proto-oncogene MYC which binds to the promoter region directly upstream of the miR-17 locus [40]. The cluster is highly expressed in a range of hematopoietic malignancies including *MYC*-rearranged Burkitt’s lymphomas [41]. High MYC-activation is also found in triple-negative breast tumors [42, 43] which could partly explain our findings of high miR-17-92-expression in triple-negative cancers. Similarly, the miR-17-92 promoter has binding sites for HES1 [39], a transcriptional repressor in the Notch signaling pathway which is overexpressed in triple-negative breast cancer [44]. N-myc has also been found to induce miR-17-92 expression in medulloblastomas [45]. NDRG2, N-myc downstream-regulated gene 2, has been found to be significantly higher in triple-negative breast cancers compared to other subtypes [46], again indicating that differences in expression and activity of transcription factors targeting the miR-17-92 promoter vary between breast cancer subtypes and can explain differences in miR-17-92 expression.

However, we also observed significant changes in the expression of miRNAs that are members of the paralogue cluster miR-106b-25 on chromosome 7 comprising miR-106b, miR-93 and miR-25. The miR-106b-25 cluster and its host gene, *MCM7*, as well as miR-20a in the miR-17-92 cluster, are induced by the transcription factors E2F1 and E2F3 which are regulated by MYC [47–49]. The cluster has also been shown to be regulated by bromodomain protein 4 (BRD4) which is increased in MYC-driven cancers [50]. Similar to the miR-17-92-cluster, miR-106b-25 is transcriptionally regulated by N-myc [51]. Of note, the expression of the miRNAs in the clusters is also regulated by mechanisms such as epigenetic modifications induced by hypoxia [52], independent transcription of pri-miRNAs from an alternative promoter, alternative splicing [53], and post-transcriptional modifications of the long primary transcripts based on their tertiary structure [54, 55]. These mechanisms allow for differential expression of the individual miRNAs within the clusters and miRNA families. Further, differences in expression of *DICER1*, *AGO* and *DROSHA,* all crucial to miRNA biosynthesis, between breast cancer subtypes have been shown [28]. Summarized, the paralogous clusters seem to have important transcription factors and regulatory pathways in common. Indeed, it has been shown that the clusters are evolutionary conserved and it is suggested that they derive from a single gene that underwent duplication, mutations and losses of individual miRNAs [56, 57].

Further, clustered miRNAs seem to cooperate by regulating similar sets of genes belonging to specific signaling pathways [58] which fits with the sequence homology and conserved seed sequences of the miRNA within the clusters [59]. The miR-17 family of miRNAs share the same seed sequence of special importance for binding and targeting mRNAs, and include miR-17-5p, miR-20a-5p, miR-20b-5p, miR-106a-5p, miR-106b-5p and miR-93-5p from clusters miR-17-92, 106b-25 and the third paralogue cluster miR-106a-363 [38]. Of the three paralogues, involving four miRNA families, the miR-17-92 cluster is best described. MiR-17 and miR-19a have been shown to target mitogen activated kinases (MAPKs) such as extracellular signal-regulated kinase (ERK) 1/2, and key signaling molecules in the MAPK signaling pathway such as KRAS and RAF1 [60]. The MAPK signaling pathways regulate cellular proliferation, migration, differentiation and cell death and are dysregulated in many cancers, including breast cancer [61]. Note that both miR-17 and miR-20a have been shown to target E2F1, thereby taking part in a negative feed-back loop where the E2F transcription factors induce transcription of miRNAs that have the same transcription factors as their target [40, 49]. In addition, miR-17 and miR-20a targets the type II transforming growth factor β (TGF-β) receptor II whereas miR-18a targets Smad 4 downstream in the TGF-β signaling pathway, thereby opposing the tumor-suppressive effects of TGF-β and promoting angiogenesis [62]. Interestingly, miR-17-92 has also been found to target the cyclin-dependent kinase inhibitor p21 and the apoptosis facilitator BCL2L11 which are mediators of TGF-β effects on proliferation and apoptosis [57]. Noteworthy, BCL2L11 is also targeted by miR-106b-25, again underlining how the miRNA clusters share targets [63]. MiR-17-5p has been shown in cellular assays to play an important role in cancer cell invasion and migration by suppressing HBP1 and consequently Wnt/β-catenin [64]. The miR-17-92 cluster members can also suppress the specificity protein (Sp) repressor ZBTB4, which in turn facilitates upregulation of Sp transcription factors and their target genes, thereby displaying tumor promoting functions [65]. MiR-19 has been shown to excert oncogenic activity through binding to and repression of the tumor suppressor PTEN and activation of the Akt-mTOR (mammalian target of rapamycin) pathway to promote cell survival [66]. Further, the tumor suppressor p53 is a direct target of miR-25 [67]. MiR-25 has also been shown to promote proliferation in triple-negative breast cancer cells by repression of the BTG anti-proliferation factor 2 [68] whereas miR-106b has been shown to induce proliferation by targeting RB proteins in various cancers [69, 70]. MiR-93 has been shown to increase proliferation, migration and invasion potential of MCF7 breast cancer cells and to have many potential targets involved in tumor growth, including the large tumor suppressor homologue 2 (LATS2) [33]. In summary, the miR-17-92 cluster and the miR-17 family of miRNAs have been demonstrated to regulate functions at the very core of malignancy: invasion, metastasis, cellular proliferation and resistance to apoptosis.

Microarray miRNA profiling of human breast cancer has been demonstrated to be an informative tool which can be used to classify human breast cancers [19]. However, interpretation of research data, and implementation of miRNA-profiling into clinical practice are complicated by the apparent lack of consistency between studies. Variation in study size, design and experimental factors such as sample type, RNA quality, methods and technology platform used is challenging when trying to summarize clinically relevant data on miRNA profiling. Further, investigated miRNAs are not identical between studies. However, this study using FFPE-tissue, immunohistochemical analyses used in everyday diagnostics and a microarray with 94% coverage of human miRNAs, indicates that the miR-17-92 cluster and miR-17-family of miRNAs are of special interest in the high grade, triple-negative tumors with the worst prognosis. Further studies, including validation in an independent cohort, in situ hybridization in tissues and analyses of prediagnostic blood samples could be of interest to further evaluate the miRNA expression within tumor cells, the tumors’ microenvironment and in the circulation, and the potential of these miRNAs in diagnostic pathology and clinical oncology.

## Conclusions

In the NOWAC postgenome cohort, archived FFPE tissue was found suitable for miRNA analyses using microarray and PCR technology. We found the miRNA expression to be significantly different between benign and malignant breast tissue. When exploring the miRNA expression profiles according to receptor status, histologic grade and molecular subtypes, we found a coordinated clear upregulation of miR-17-5p, miR-20a-5p, miR-92a-3p, miR-106b-5p and miR-93-5p of the miR-17-92 cluster and the miR-17-family in the most aggressive tumors with higher tumor grade and triple-negative receptor status. A validation of this pilot study in an independent cohort is a focus for future research. Further, the diagnostic and functional role of the miR-17-family of miRNAs in breast cancer should be further explored, where miRNAs’ potential as markers of response to specific treatments or as biomarkers of breast cancer relapse or metastatic disease is highly interesting.

## Supplementary information

**Additional file 1: Table S1.** MiRNAs demonstrating significantly different expression according to tumor size. Expression levels are given as the mean (standard deviation) of log2 transformed intensity values (Hy3) by microarray-analyses.*False discovery rate (FDR) adjusted *p*-value. E indicates exponential number.

## Data Availability

The datasets analyzed during the current study are available from the European Genome-phenome Archive (Dataset ID: EGAD00010001406).

## Abbreviations

ER: estrogen receptor
PR: progesterone receptor
FFPE: formalin-fixed paraffin-embedded
miRNA: microRNA
NOWAC: Norwegian Women and Cancer
FDR: false discovery rate
