# Supplementary material for: Differential expression of the miR-17-92 cluster and miR-17 family in breast cancer according to tumor type; results from the Norwegian Women and Cancer (NOWAC) study
Source: J Transl Med. 2019 Oct 3;17:334. doi: 10.1186/s12967-019-2086-x (PMC6775665; doi:10.1186/s12967-019-2086-x)
Supplement: Supplementary file 1 — Additional file 1: Table S1. MiRNAs demonstrating significantly different expression according to tumor size. Expression levels are given as the mean (standard deviation) of log2 transformed intensity values (Hy3) by microarray-analyses.*False discovery rate (FDR) adjusted p-value. E indicates exponential number. [file 12967_2019_2086_MOESM1_ESM.docx]

| MiRNA | TUMOR  ≤ 20 MM |  | TUMOR  > 20 MM |  |  |
| --- | --- | --- | --- | --- | --- |
|  | **Mean ± SD** |  | **Mean ± SD** | **logFC** | ***P**** |
| miR-2964a-5p  miR-4290  miR-548k  miR-h6-3p  let-7d-5p  miR-1184  miR-30b-5p  miR-98-5p  let-7f-5p  miR-489  miR-4728-3p  miR-4291  miR-195-5p  miR-26b-5p  miR-4467  miR-365a-3p  miR-193a-3p  miR-101-3p  miR-143-3p  miR-183-3p  miR-126-5p  miR-145-5p  miR-4328  miR-10b-5p  miR-4324  miR-24-3p  miR-553  miR-943  miR-199b-5p  miR-30c-5p  miR-99a-5p  miR-221-5p  miR-1297  miR-k12-3-5p  miR-25-5p  miR-615-3p  let-7a-2-3p  miR-338-5p  mir-371b-5p  miR-519e-5p  miR-100-5p  miR-3152-5p  miR-3124-3p  miR-2355-3p  miR-4778-3p  miR-551a  miR-4478  miR-3164  miR-342-5p  miR-126-3p  miR-2113  miR-3960  miR-bart13-3p  miR-26a-5p  miR-224-3p  miR-877-5p  miR-125a_5p  miR-4279  miR-99b-5p  miRplus-a1015  miR-3158-5p  miR-k12-5-5p  miR-29a-3p  miR-30d-5p  miR-29b-3p  miR-130a-3p  miR-107  miR-205-5p  miR-766-5p  let-7i-5p  miR-133b  miR-4308  miR-4419b  miR-23a-3p  miR-642b-3p  miR-30e-3p  miR-29c-3p  miRplus-j1003  miR-552  miR-27a-3p  miR-5196-3p  miR-4329  miR-4787-5p  miR-30e-5p  miR-1264  miR-30a-5p  miR-23b-3p  miR-4317  miR-150-5p  miR-548ap-5p  let-7a-5p  miR-4682  miR-4768-5p  miR-498  miR-3148  let-7c  let-7g-5p  miR-4500  miR-h25  miR-3686  miR-4450  miR-27b-3p  miR-204-3p  miR-151a-5p  miR-181a-2-3p  miR-15a-5p  miR-199a-3p  miR-10a-5p  miR-4795-3p  miR-585  miR-423-5p  miR-548t-5p  miR-bart15  miR-4484  miR-3911  miR-485-3p  miR-711  miR-4436b-3p  miR-22-3p  miR-125b-5p  let-7d-3p  miR-1248  miR-h9-3p  miR-4502  miR-140-3p  miR-4455  miR-3654  miR-516b-5p  miR-4311  miR-920  miR-451a  let-7e-5p  miR-3621  miR-4762-5p  miR-1268b  miR-20a-5p  miR-4708-3p  miR-574-3p  miR-92a-3p  miR-5004-5p  miR-4685-3p  miR-15b-5p  miR-584-5p  miR-675-5p  miR-513b  mir-934  snord38b  miR-124-5p  miR-642b-5p  let-7b-5p  miR-29b-1-5p  miR-2392  miR-k12-6-3p  miR-1273f  miR-4784  miR-16-5p  miR-1246  miR-bhrf1-2-3p  miR-22-5p  miR-4732-3p  miR-5096  miR-141-5p  miR-645  miR-1827  miR-302a-3p  miR-bart16  miR-1306-5p  miR-513a-5p  miR-4425  miR-4443  miR-4301  miR-1247-5p  miR-221-3p  miR-378a-3p  miR-h7-3p  miR-298  miR-5002-5p  miR-4788  miR-5095  miR-3611  miR-4667-5p  snord12  miR-4674  miR-4540  miR-3664-5p  miR-34a-5p  miR-103a-3p  miR-767-5p  miR-4711-3p  miR-519d  miR-4644  miR-5585-3p  miR-3679-3p  miR-4800-3p  miR-1298  miR-3660  miR-4795-5p  miR-4491  miR-4436b-5p  miR-3687  miR-196a-5p  miR-4451  miR-4472  miR-921  miR-4297  miR-638  miR-205-3p  miR-892a  miR-222-3p | 11.50 ± 0.51  12.05 ± 0.20  5.99 ± 0.18  12.20 ± 0.18  7.61 ± 0.44  7.16 ± 0.30  7.37 ± 0.59  6.80 ± 0.26  6.41 ± 0.22  6.67 ± 0.28  8.66 ± 0.24  6.10 ± 0.22  7.07 ± 0.50  7.73 ± 0.53  13.07 ± 0.44  6.92 ± 0.35  6.13 ± 0.34  5.98 ± 0.29  7.69 ± 0.46  6.93 ± 0.25  6.17 ± 0.19  6.89 ± 0.48  7.03 ± 0.53  7.02 ± 0.39  7.34 ± 0.43  8.14 ± 0.46  6.55 ± 0.13  8.47 ± 0.28  6.25 ± 0.28  7.90 ± 0.63  6.91 ± 0.64  7.05 ± 0.54  6.28 ± 0.147  8.39 ± 0.31  8.06 ± 0.27  6.95 ± 0.18  6.53 ± 0.12  6.92 ± 0.58  12.79 ± 0.41  7.09 ± 0.21  6.20 ± 0.36  6.96 ± 0.30  12.05 ± 0.16  8.09 ± 0.28  6.79 ± 0.30  6.53 ± 0.13  8.44 ± 0.22  7.41 ± 0.36  6.59 ± 0.45  7.50 ± 0.47  7.89 ± 0.30  13.80 ± 0.75  7.44 ± 0.28  6.49 ± 0.51  7.12 ± 0.44  6.79 ± 0.13  8.85 ± 0.51  11.89 ± 0.17  6.61 ± 0.40  7.87 ± 0.26  7.52 ± 0.32  9.89 ± 0.80  7.24 ± 0.85  7.17 ± 0.53  6.69 ± 0.53  6.51 ± 0.44  6.73 ± 0.28  8.54 ± 1.32  6.95 ± 0.70  7.62 ± 0.39  6.56 ± 0.45  6.82 ± 0.26  11.26 ± 0.26  8.74 ± 0.57  6.16 ± 0.22  6.31 ± 0.43  6.83 ± 0.70  7.05 ± 0.31  7.20 ± 0.33  7.01 ± 0.60  7.54 ± 0.23  6.77 ± 0.19  13.65 ± 0.72  6.20 ± 0.37  7.26 ± 0.30  6.47 ± 0.63  8.61 ± 0.60  6.58 ± 0.19  8.80 ± 0.35  8.99 ± 0.50  9.07 ± 0.77  7.58 ± 0.41  6.75 ± 0.43  8.06 ± 0.23  8.48 ± 0.23  8.22 ± 0.68  8.28 ± 0.50  8.67 ± 0.36  9.15 ± 0.48  11.64 ± 0.24  7.37 ± 0.19  6.65 ± 0.56  9.94 ± 0.49  6.19 ± 0.50  6.20 ± 0.34  6.93 ± 0.35  7.44 ± 0.63  7.20 ± 0.73  8.95 ± 0.47  8.70 ± 0.43  8.62 ± 0.24  6.69 ± 0.26  6.14 ± 0.20  7.48 ± 0.38  6.63 ± 0.10  7.87 ± 0.21  6.93 ± 0.21  6.90 ± 0.34  7.75 ± 0.52  9.85 ± 0.96  7.08 ± 0.18  7.16 ± 0.28  6.51 ± 0.23  6.82 ± 0.28  6.25 ± 0.37  10.56 ± 0.36  7.55 ± 0.26  7.22 ± 0.16  6.40 ± 0.12  7.11 ± 0.19  7.11 ± 1.10  8.96 ± 0.25  7.30 ± 0.31  6.87 ± 0.28  7.01 ± 0.36  6.67± 0.63  14.29 ± 0.87  7.43 ± 0.19  7.22 ± 0.53  7.02 ± 0.35  6.62 ± 0.25  6.93 ± 0.45  7.03 ± 0.19  7.57 ± 0.35  6.98 ± 0.25  6.56 ± 0.14  8.28 ± 0.37  6.33 ± 0.18  12.17 ± 0.19  10.08 ± 0.85  6.92 ± 0.29  6.59 ± 0.25  9.48 ± 0.49  7.90 ± 0.29  8.15 ± 0.19  8.38 ± 0.73  12.36 ± 0.30  9.82 ± 0.57  7.21 ± 0.28  8.40 ± 0.25  6.15 ± 0.26  6.23 ± 0.28  6.53 ± 0.39  9.67 ± 0.31  10.39 ± 0.46  8.25 ± 0.23  6.37 ± 0.10  10.17 ± .48  6.74 ± 0.36  12.37 ± 0.15  10.98 ± 0.54  6.76 ± 0.16  6.74 ± 0.33  7.09 ± 0.22  9.41 ± 0.42  6.50 ± 0.17  9.17 ± 0 .35  7.53 ± 0 .37  7.12 ± 0.24  8.23 ± 0.24  9.12 ± 0.37  7.17 ± 0.36  6.42 ± 0.24  7.67 ± 0.23  6.70 ± 0.31  7.15 ± 0.50  7.65 ± 0.42  6.94 ± 0.28  7.01 ± 0.27  6.33 ± 0.19  9.56 ± 0.46  7.03 ± 0.28  7.66 ± 0.33  14.36 ± .88  6.37 ± 0.19  6.24 ± 0.18  9.70 ± 0.41  6.83 ± 0 .51  8.03 ± 0.17  8.54 ± 0.57  6.80 ± 0.68  7.39 ± 0.17  8.75 ± 0 .38 7.82 ± 0.42  7.47 ± 0.26  9.35 ± 0.43  8.15 ± 0.22  6.70 ± 0.12  7.10 ± 0.51 |  | 9.00 ± 2.78  12.69 ± 0.78  6.44 ± 0.59  12.84 ± 0.88  8.40 ± 1.06  7.74 ± 0.78  8.26 ± 1.14  7.43 ± 0.87  6.99 ± 0.82  7.21 ± 0.75  8.93 ± 0.31  6.45 ± 0.49  8.07 ± 1.43  8.64 ± 1.28  13.73 ± 0.90  7.41 ± 0.69  6.57 ± 0.61  6.60 ± 0.92  8.67 ± 1.46  7.45 ± 0.78  6.61 ± 0.67  7.82 ± 1.39  7.99 ± 1.42  7.74 ± 1.07  8.11 ± 1.16  8.84 ± 1.00  6.41 ± 0.19  8.80 ± 0.46  6.92 ± 1.04  8.65 ± 1.04  7.89 ± 1.47  6.57 ± 0.58  6.63 ± 0.55  8.90 ± 0.78  8.50 ± 0.67  6.76 ± 0.27  6.77 ± 0 .39  6.45 ± 0.55  13.54 ± 1.20  7.51 ± 0.68  6.80 ± 0.96  6.68 ± 0.39  12.65 ± 1.03  7.84 ± 0.34  6.51 ± 0.40  6.79 ± 0.44  8.06 ± 0.63  6.78 ± 1.06  6.23 ± 0.46  8.28 ± 1.34  8.28 ± 0.64  14.46 ± .93  7.82 ± 0.62  7.14 ± 1.06  6.78 ± 0.45  7.05 ± 0.44  9.40 ± 0.88  12.29 ± 0.72  7.02 ± 0.64  8.31 ± 0.78  8.05 ± 0.93  9.26 ± 0.87  8.11 ± 1.40  7.71 ± 0.85  7.36 ± 1.16  7.15 ± 1.11  6.98 ± 0.38  9.62 ± 1.60  6.47 ± 0.61  8.08 ± 0 .80  6.24 ± 0.41  6.62 ± 0.28  10.75 ± 0.96  9.32 ± 1.00  6.40 ± 0.42  6.70 ± 0.64  7.51 ± 1.17  6.81 ± 0.38  7.62 ± 0.79  7.57 ± 0.98  7.27 ± 0 .50  7.02 ± 0.48  14.20 ± .87  6.58 ± 0 .72  7.67 ± 0.80  7.00 ± 0.93  9.18 ± 1.04  6.78 ± 0.36  9.15 ± 0.64  9.44 ± 0.81  9.66 ± 1.00  7.26 ± 0.53  6.47 ± 0.43  8.26 ± 0.37  8.26 ± 0.43  8.72 ± 0.86  8.78 ± 0.98  9.09 ± 0.85  9.55 ± 0.74  12.19 ± 1.19  7.18 ± 0.38  7.12 ± 0.88  10.36 ± 0.81  6.56 ± 0.65  6.49 ± 0.57  7.25 ± 0.64  8.02 ± 1.17  7.74 ± 1.01  9.41 ± 0.96  8.28 ± 0.89  8.86 ± 0.50  6.51 ± 0.33  6.37 ± 0.50  7.83 ± 0.72  6.53 ± 0.21  7.69 ± 0.35  7.17 ± 0.52  6.69 ± 0.38  8.25 ± 1.08  10.67 ± 1.72  7.25 ± 0.37  7.39 ± 0.50  6.67 ± 0.33  6.64 ± 0.32  6.57 ± 0.71  10.28 ± 0.61  7.77 ± 0.51  7.37 ± 0.35  6.32 ± 0.16  6.98 ± 0.26  8.03 ± 2.08  9.16 ± 0.47  7.52 ± 0.50  7.12 ± 0.57  6.81 ± 0.39  7.04 ± 0.72  14.77 ± 0.94  7.58 ± 0.35  7.53 ± 0.62  6.79 ± 0.50  6.45 ± 0.38  7.19 ± 0.54  7.19 ± 0.38  7.83 ± 0.58  7.20 ± 0.52  6.68 ± 0.29  8.60 ± 0.78  6.49 ± 0.41  12.37 ± 0.52  10.51 ± 0.82  6.75 ± 0.37  6.46 ± 0.28  9.78 ± 0.71  7.70 ± 0.49  7.99 ± 0.41  8.81 ± 1.00  12.73 ± 1.05  10.13 ± 0.71  6.98 ± 0.64  8.21 ± 0.52  6.30 ± 0.37  6.44 ± 0.56  6.36 ± 0.35  9.83 ± 0.37  10.16 ± 0.54  8.09 ± 0.45  6.31 ± 0.14  10.43 ± 0.66  6.95 ± 0.53  12.61 ± 0.73  10.67 ± 0.80  6.86 ± 0.27  6.94 ± 0.55  7.27 ± 0.54  9.59 ± 0.40  6.41 ± 0.19  9.43 ± 0.72  7.30 ± 0.60  6.97 ± 0.39  8.43 ± 0.58  9.38 ± 0.76  7.03 ± 0.26  6.56 ± 0.36  7.52 ± 0.41  6.53 ± 0.45  7.40 ± 0.67  7.87 ± 0.55  6.82 ± 0.27  6.88 ± 0.36  6.22 ± 0.32  9.32 ± 0.64  6.88 ± 0.41  7.45 ± 0.64  14.80 ± 1.21  6.24 ± 0.40  6.35 ± 0.32  9.47 ± 0.67  7.09 ± 0.72  7.94 ± 0.25  8.29 ± 0.63  6.54 ± 0.58  7.52 ± 0.41  8.54 ± 0.59  8.05 ± 0.66  7.36 ± 0.29  9.58 ± 0.66  8.02 ± 0.39  6.60 ± 0.32  7.36 ± 0.74 | -2.50  0.64  0.46  0.64  0.79  0.58  0.89  0.63  0.58  0.55  0.26  0.35  1.00  0.91  0.66  0.50  0.45  0.62  0.98  0.93  0.69  0.93  0.96  0.72  0.78  0.96  -0.14  0.33  0.67  0.74  0.98  -0.48  0.35  0.51  0.44  -0.19  0.25  -0.48  0.75  0.42  0.60  -0.29  0.60  -0.25  -0.28  0.26  -0.38  -0.63  -0.36  0.79  0.39  0.66  0.37  0.65  -0.35  0.25  0.56  0.41  0.41  0.44  0.53  -0.63  0.88  0.53  0.68  0.64  0.25  1.08  -0.48  0.46  -0.32  -0.20  -0.51  0.58  0.24  0.39  0.68  -0.24  0.42  0.56  -0.27  0.25  0.55  0.39  0.41  0.53  0.57  0.20  0.35  0.45  0.59  -0.31  -0.28  0.20  -0.22  0.50  0.50  0.42  0.40  0.55  -0.19  0.47  0.42  0.36  0.30  0.32  0.58  0.55  0.46  -0.43  0.24  -0.18  0.23  0.35  -0.10  -0.17  0.24  -0.21  0.50  0.82  0.17  0.23  0.16  -0.17  0.32  -0.28  0.23  0.15  -0.08  -0.13  0.92  0.21  0.23  0.25  -0.20  0.36  0.48  0.15  0.31  -0.22  -0.17  0.26  0.15  0.25  0.22  0.12  0.32  0.16  0.20  0.42  -0.17  -0.13  0.30  -0.20  -0.16  0.42  0.37  0.31  -0.23  -0.20  0.15  0.21  -0.17  0.16  -0.23  -0.17  -0.06  0.27  0.21  0.24  -0.31  0.10  0.21  0.19  0.19  -0.08  0.26  -0.22  -0.15  0.20  0.27  -0.14  0.14  -0.15  -0.17  0.26  0.21  -0.11  -0.14  -0.11  -0.24  -0.15  -0.22  0.45  -0.13  0.11  -0.23  0.26  -0.09  -0.25  -0.26  0.13  -0.20  0.23  -0.11  0.23  -0.13  -0.10  0.26 | 1.26E-08  2.55E-07  1.58E-06  4.01E-06  4.90E-06  4.90E-06  4.90E-06  5.59E-06  5.59E-06  5.79E-06  6.70E-06  9.03E-06  9.03E-06  9.03E-06  9.03E-06  1.28E-05  1.28E-05  1.34E-05  1.34E-05  1.41E-05  1.41E-05  1.41E-05  1.41E-05  1.41E-05  1.41E-05  1.41E-05  1.61E-05  1.68E-05  1.68E-05  2.26E-05  2.26E-05  2.60E-05  2.70E-05  2.70E-05  2.86E-05  3.24E-05  3.62E-05  3.62E-05  4.08E-05  4.53E-05  5.01E-05  5.97E-05  6.33E-05  6.78E-05  7.16E-05  7.16E-05  7.16E-05  9.50E-05  9.70E-05  1.03E-04  1.03E-04  1.03E-04  1.03E-04  1.03E-04  1.05E-04  1.07E-04  1.17E-04  1.17E-04  1.19E-04  1.46E-04  1.50E-04  1.51E-04  1.62E-04  1.73E-04  1.82E-04  1.82E-04  1.82E-04  2.05E-04  2.27E-04  2.27E-04  2.27E-04  3.23E-04  3.28E-04  3.68E-04  3.99E-04  4.03E-04  4.13E-04  4.47E-04  5.06E-04  5.37E-04  5.44E-04  5.57E-04  5.85E-04  7.66E-04  7.66E-04  7.66E-04  7.72E-04  8.40E-04  8.48E-04  9.54E-04  9.55E-04  1.02E-03  1.16E-03  1.16E-03  1.30E-03  1.32E-03  1.32E-03  1.32E-03  1.34E-03  1.39E-03  1.45E-03  1.51E-03  1.57E-03  1.63E-03  1.80E-03  1.86E-03  1.91E-03  1.97E-03  2.21E-03  2.34E-03  2.50E-03  2.64E-03  2.69E-03  2.81E-03  2.85E-03  2.89E-03  3.10E-03  3.21E-03  3.43E-03  3.43E-03  4.48E-03  4.79E-03  4.79E-03  5.10E-03  5.10E-03  5.28E-03  5.49E-03  5.73E-03  5.81E-03  5.96E-03  6.28E-03  6.37E-03  6.82E-03  6.82E-03  7.64E-03  7.73E-03  7.95E-03  8.13E-03  8.16E-03  8.80E-03  8.80E-03  8.80E-03  8.80E-03  8.80E-03  8.83E-03  9.53E-03  1.01E-02  1.11E-02  1.14E-02  1.14E-02  1.22E-02  1.42E-02  1.50E-02  1.51E-02  1.52E-02  1.84E-02  1.98E-02  2.02E-02  2.10E-02  2.10E-02  2.19E-02  2.19E-02  2.23E-02  2.23E-02  2.24E-02  2.38E-02  2.39E-02  2.40E-02  2.46E-02  2.58E-02  2.58E-02  2.59E-02  2.65E-02  2.65E-02  2.65E-02  2.65E-02  2.83E-02  2.86E-02  2.99E-02  2.99E-02  3.10E-02  3.16E-02  3.17E-02  3.20E-02  3.22E-02  3.29E-02  3.33E-02  3.33E-02  3.38E-02  3.74E-02  3.82E-02  3.83E-02  3.85E-02  3.88E-02  3.88E-02  4.09E-02  4.11E-02  4.42E-02  4.42E-02  4.49E-02  4.49E-02  4.51E-02  4.51E-02  4.53E-02  4.53E-02  4.64E-02  4.83E-02  4.86E-02  4.86E-02 |
